# Association of coping strategies with mortality and health-related quality of life in hemodialysis patients: The Japan Dialysis Outcomes and Practice Patterns Study

**DOI:** 10.1371/journal.pone.0180498

**Published:** 2017-07-25

**Authors:** Kakuya Niihata, Shingo Fukuma, Tadao Akizawa, Shunichi Fukuhara

**Affiliations:** 1 Division of Clinical Epidemiology, Fukushima Medical University, Fukushima, Japan; 2 Center for Innovative Research for Communities and Clinical Excellence (CIRC^2^LE), Fukushima Medical University, Fukushima, Japan; 3 Department of Healthcare and Epidemiology, School of Public Health, Kyoto University of Medicine, Kyoto, Japan; 4 Institute for Health Outcomes and Process Evaluation Research (iHope International), Kyoto, Japan; 5 Division of Nephrology, Department of Medicine, Showa University School of Medicine, Tokyo, Japan; King's College London, UNITED KINGDOM

## Abstract

**Background:**

Hemodialysis patients are exposed to disease- and treatment-related stresses, and use various coping strategies to deal with these stresses. Although some studies have reported the association of coping strategies with mortality or health-related quality of life (QOL) in some populations, the effect of coping strategies on clinical outcomes in hemodialysis patients remains unclear. We investigated the association in a longitudinal design among Japanese hemodialysis patients.

**Methods:**

We examined Japanese hemodialysis patients who participated in the Dialysis Outcomes and Practice Patterns Study (DOPPS) IV, which was conducted between 2009 and 2012. The exposure variable was stress coping strategy, as assessed using subscales in Coping Strategies Inventory Short Form: problem-focused engagement, problem-focused disengagement, emotion-focused engagement, and emotion-focused disengagement. Hazard ratios were estimated using Cox proportional hazard model for all-cause mortality and mean differences for change in health-related QOL in 1 year were estimated using a regression model.

**Results:**

Among 1,354 patients, only problem-focused engagement was significantly associated with longer survival; other subscales were not associated with all-cause mortality after adjustment for potential confounding factors. In terms of health-related QOL, the subscale of problem-focused engagement was also associated with improvement in physical functioning and mental health among 1,045 patients. Emotion-focused disengagement was associated with deterioration in mental health, but not with change in physical functioning. The other subscales were not associated with change in physical functioning or mental health.

**Conclusions:**

Among hemodialysis patients, “problem-focused engagement” coping strategies were associated with longer survival and also with improvement in physical functioning and mental health. To achieve greater longevity and improve QOL in hemodialysis patients under ongoing stresses, problem-focused engagement should be encouraged.

## Introduction

Hemodialysis patients are exposed to chronic stresses due to their specific symptoms and treatments, including itching, fatigue, and fluid and diet restrictions. In addition, hemodialysis patients usually need to receive dialysis therapy three times weekly, which places limitations on their social life[1,2]. Certain coping strategies may alleviate these stresses[3]. Among several other populations, such as patients with cancer or chronic heart failure, some coping strategies have been reported to be associated with improvements in clinical outcomes, including mortality and health-related quality of life[4–6]. We hypothesized that coping strategies could also influence outcomes for hemodialysis patients, and have therefore sought to clarify the association between coping strategies and clinical outcomes.

Coping strategies are applied as cognitive and behavioral processes for stress management in individuals. Coping strategies have been categorized into broad strategies, such as problem-focused coping strategies and emotion-focused coping strategies. Problem-focused coping strategies, including seeking social support, planning, and problem solving, are believed to be more adaptive processes to modify stressful situations. On the other hand, emotion-focused coping strategies, including escape-avoidance, behavioral disengagement, venting, and denial, are believed to be more maladaptive processes for individuals to give up modifying situations in order to alleviate stress[6]. Coping strategies have been believed to be relatively stable over time[7]; however, recent reports suggest that coping strategies could be modifiable through cognitive behavioral therapies[8–10]. Cognitive therapies are reported to be effective in hemodialysis patients with depressive symptoms[11]; however, the kind of coping strategies that should be recommended for hemodialysis patients is unclear.

In several populations, it has been reported that coping strategies are associated with relevant clinical outcomes. Though the associations between coping strategies and mortality remain controversial[12], several studies have reported that emotion-focused coping strategies, such as expression of emotion, were associated with decreased mortality in cancer patients[4,5,13]. In a population of patients with chronic heart failure, a previous study reported that coping strategies were associated with health-related quality of life (HR-QOL)[6]. Problem-focused coping strategies, such as problem solving, were associated with improved physical and psychological HR-QOL, while emotion-focused coping strategies, such as escape-avoidance or behavioral disengagement, were associated with reduced HR-QOL. The associations between coping strategies and clinical outcomes have also been investigated in hemodialysis patients. The emotion regulation strategy in hemodialysis patients has been reported to have significant implications for attitude towards the disease[7], whereas another study reported that avoidant coping was associated with increased mortality among 61 veterans with end-stage renal disease (ESRD)[14]. Despite these efforts, the association between coping strategies and clinical outcomes in hemodialysis patients remains unclear because most of these previous studies used cross-sectional designs and small sample sizes.

Here, we evaluated the association between coping strategies and relevant clinical health outcomes, including mortality and HR-QOL, in hemodialysis patients using a longitudinal study design.

## Subjects and methods

### Design and setting

The Dialysis Outcomes and Practice Patterns Study (DOPPS) is an international prospective cohort study of outcomes and practice patterns for hemodialysis patients. The sampling method of the study participants applied in DOPPS has been previously described in detail[15,16]. In the present study, we analyzed the data of 2,742 participants from the Japanese DOPPS (J-DOPPS) IV, which was conducted between March 1^st^, 2009 and February 29^th^, 2012. All participants provided written informed consent. The study complied with the Declaration of Helsinki, and was approved by the Ethics Committee of Kyoto University Graduate School of Medicine.

### Study population

Eligible participants were patients aged ≥18 years who were on maintenance hemodialysis therapy for ≥3 months. Patients with missing exposure or outcomes data for this study were excluded.

### Exposure

**Fig 1:**
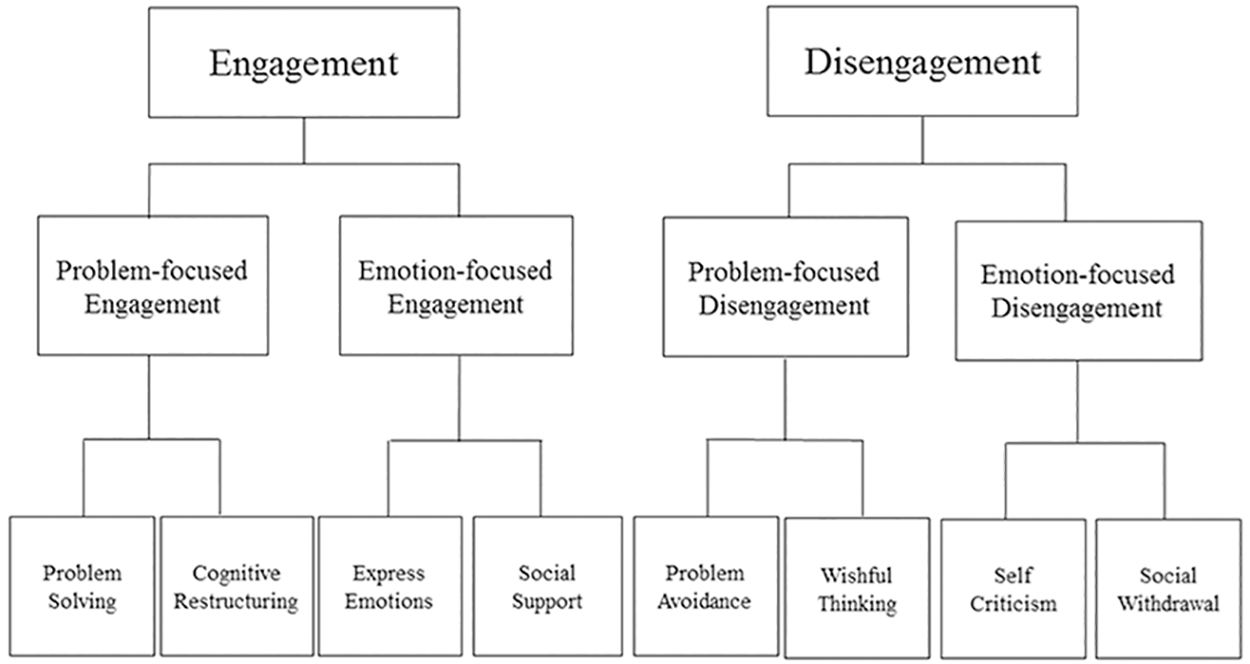
Hierarchical structure of the CSI.

The main exposure variable was stress coping strategy, as assessed using the Coping Strategies Inventory Short Form (CSI-SF) (Table 1). The CSI-SF is a 16-item questionnaire that was shortened from the original Coping Strategies Inventory (CSI) and validated in African Americans[17]. We used the CSI-SF translated into Japanese. The structure of the original CSI is shown in Fig 1[18]. In the original CSI, coping strategies are first categorized into engagement and disengagement strategies[18]. Engagement strategies focus on approaching stressful situations to manage stress, whereas disengagement strategies focus on avoiding exposure to stressful situations to alleviate stress[17]. These two categories are further categorized into problem-focused and emotion-focused coping strategies. Problem-focused coping involves management of stressors, whereas emotion-focused coping involves effective control of individual feeling invoked by stressors[17]. The structure of CSI-SF reflects the following four subscales in the original scale: problem-focused engagement, problem-focused disengagement, emotion-focused engagement, and emotion-focused disengagement. Each subscale includes four items, and all were evaluated using a four-point Likert scale rating the frequency of coping strategies in each item as follows: 1 = “Never,” 2 = “Seldom,” 3 = “Sometimes,” 4 = “Often,” and 5 = “Almost Always.” The score of each subscale, calculated as the sum of the scores of all items in each subscale, ranged from 4 to 20. Each subscale included two dimensions[18]: problem solving and cognitive restructuring in problem-focused engagement, problem avoidance and wishful thinking in problem-focused disengagement, express emotions and social support in emotion-focused engagement, and self-criticism and social withdrawal in emotion-focused disengagement (shown in Fig 1). For example, in facing the stress of fluid restrictions, hemodialysis patients with higher problem-focused engagement drink water in a planned manner while foreseeing better outcomes, whereas those with higher problem-focused disengagement drink water without thinking about the outcomes. Those with higher emotion-focused engagement talk with their friends to express their stress or ask for advice on how to reduce it, whereas those with higher emotion-focused disengagement tend to criticize themselves if they cannot comply with restrictions without talking with their friends. We divided each subscale score into low, middle, and high groups by tertiles. We set the low group in each subscale as the reference. The associations of each subscale with the outcome were examined in separate Cox models or regression models.

**Table 1:** CSI-SF subscales.

Subscales		CSI-SF item
Problem-focused Engagement		
	#1.	I make a plan of action and follow it.
	#2.	I look for the silver lining or try to look on the bright side of things.
	#8.	I tackle the problem head on.
	#9.	I step back from the situation and try to put things into perspective.
Problem-focused Disengagement		
	#4.	I hope the problem will take care of itself.
	#7.	I try to put the problem out of my mind.
	#12.	I hope for a miracle.
	#14.	I try not to think about the problem.
Emotion-focused Engagement		
	#5.	I try to let my emotions out.
	#6.	I try to talk about it with a friend or family.
	#11.	I let my feelings out to reduce the stress.
	#13.	I ask a close friend or relative that I respect for help or advice
Emotion-focused Disengagement		
	#3.	I try to spend time alone.
	#10.	I tend to blame myself.
	#15.	I tend to criticize myself.
	#16.	I keep my thoughts and feelings to myself.

### Outcomes

The primary outcome was all-cause mortality, and the secondary outcomes were change in subscale scores of physical functioning and mental health in the short form (SF)-12[19] after 1 year. Enrolled facilities were asked to retrieve data of participants’ survival through their medical records. Patients were censored at the time of kidney transplantation, transfer to another dialysis unit, or the end of the observation period of J-DOPPS IV, February 29^th^, 2012, whichever came first.

### Statistical analysis

Baseline characteristics were presented using standard descriptive statistics: means (standard deviations) or medians (25^th^ percentiles and 75^th^ percentiles) for continuous variables and percentages for categorical variables. To compare the mortality between the participants in the final analysis and those excluded from the final analysis, a Kaplan-Meier curve was drawn using the Log-rank test. For the primary analysis, hazard ratios (HRs) and 95% confidence intervals (95% CIs) for all-cause mortality were estimated using the Cox proportional hazard model. In the Cox model, we adjusted for clinically relevant confounding factors: age, gender, years on dialysis, presence of diabetes mellitus, history of cardiovascular disease (CVD) (congestive heart failure, coronary artery disease, peripheral artery disease, cerebrovascular disease, and others), depression (defined by a score ≥10 on the Short Form of the Center for Epidemiologic Studies Depression Scale[20]), effect of kidney disease, burden of kidney disease (defined by subscales of the Kidney Disease Quality of Life [KDQOL] instrument[21]), educational status (graduated from high school), and high annual income (≥5,000,000 yen per year). For the secondary analysis, a regression model was used to estimate the mean differences and 95% CIs for changes in scores of physical functioning and mental health. In the regression model, we adjusted for baseline physical functioning and mental health scores, age, gender, years on dialysis, presence of diabetes mellitus, history of CVD, depression, effect of kidney disease, burden of kidney disease, educational status, and high annual income. A p-value of <0.05 was considered statistically significant. All analyses were performed with multiple imputation methods using STATA version 14 (Stata Corp LP, College Station, TX, USA).

### Sensitivity analysis

To examine the robustness of the association between the stress coping strategies and all-cause mortality, we conducted sensitivity analyses. The exposure and outcome variables were not retrieved from all participants of J-DOPPS IV, and excluding participants with missing data of these variables could lead to selection bias. We performed the analyses with multiple imputation for these variables. For the primary outcome, we did the same analysis as above, after multiple imputation of the exposure and outcome variables. For the secondary outcome, we performed multiple imputation with these variables, after excluding the participants who died within 1 year of enrollment.

## Results

### Baseline characteristics

**Fig 2:**
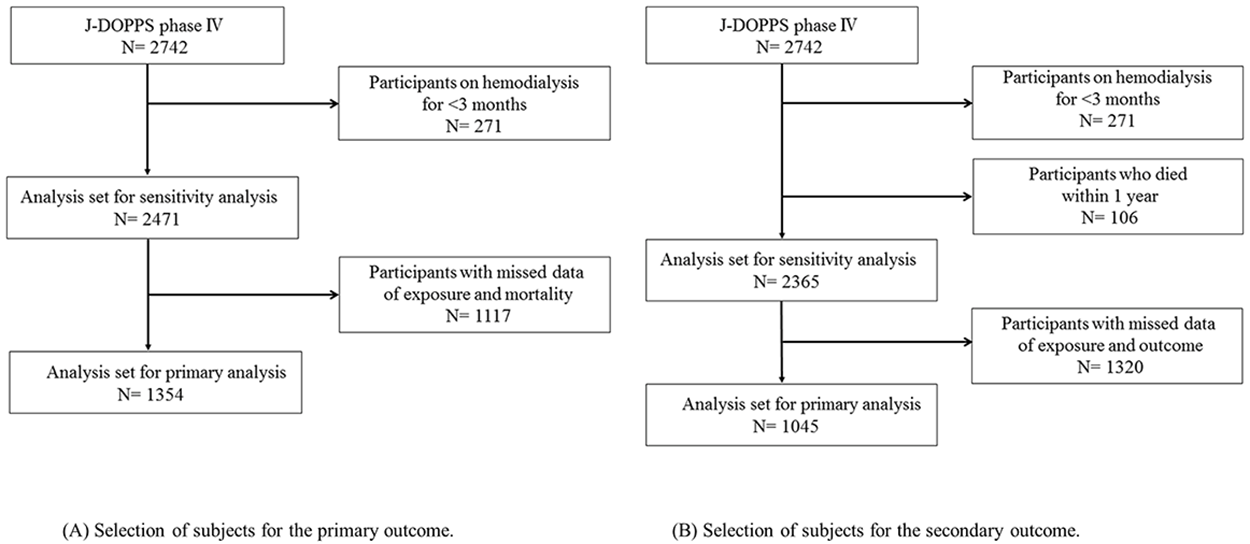
Selection of subjects. Selection of subjects for the primary outcome. (B) Selection of subjects for the secondary outcome.

**Fig 3:**
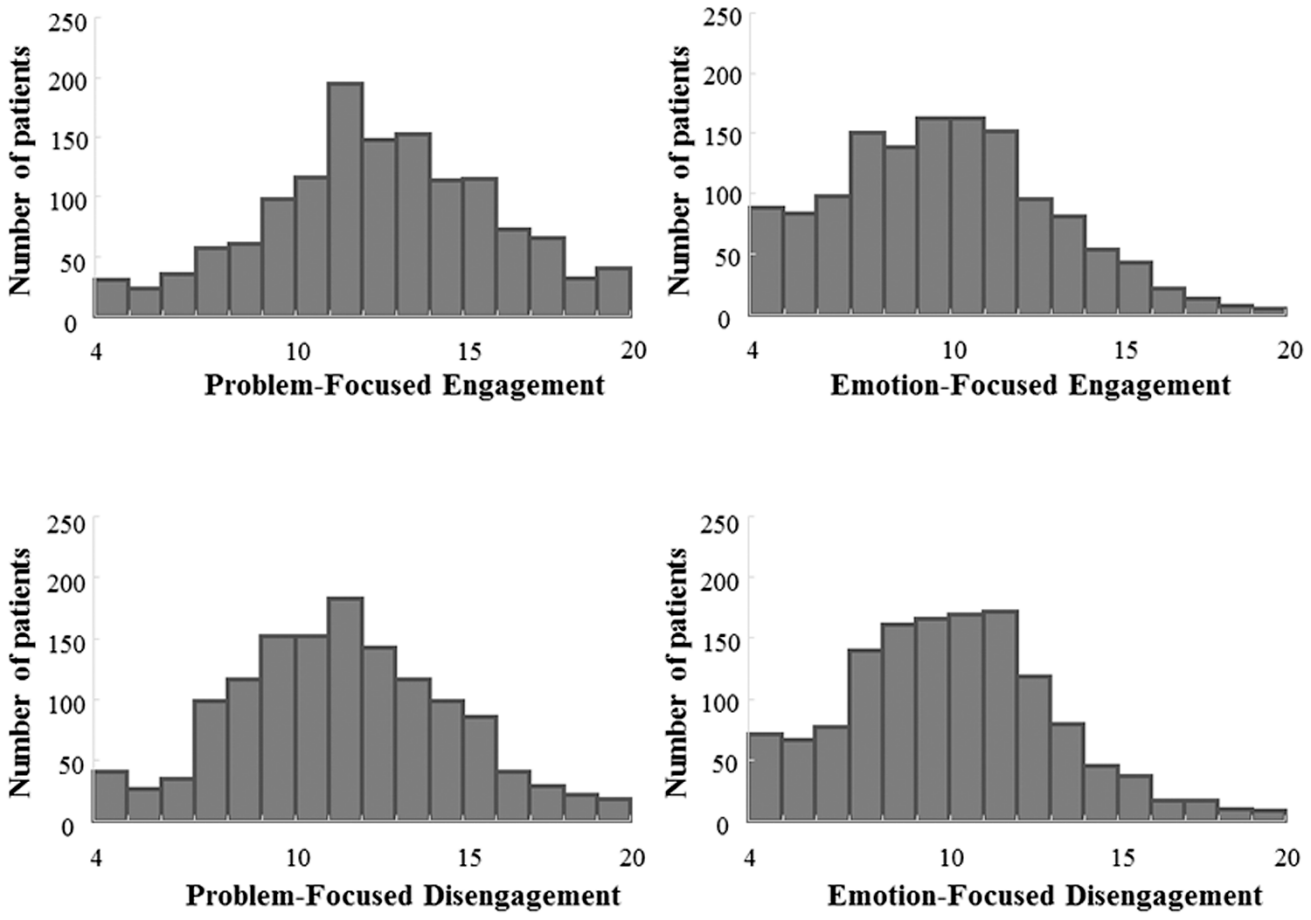
Distribution of CSI-SF subscale scores.

**Fig 4:**
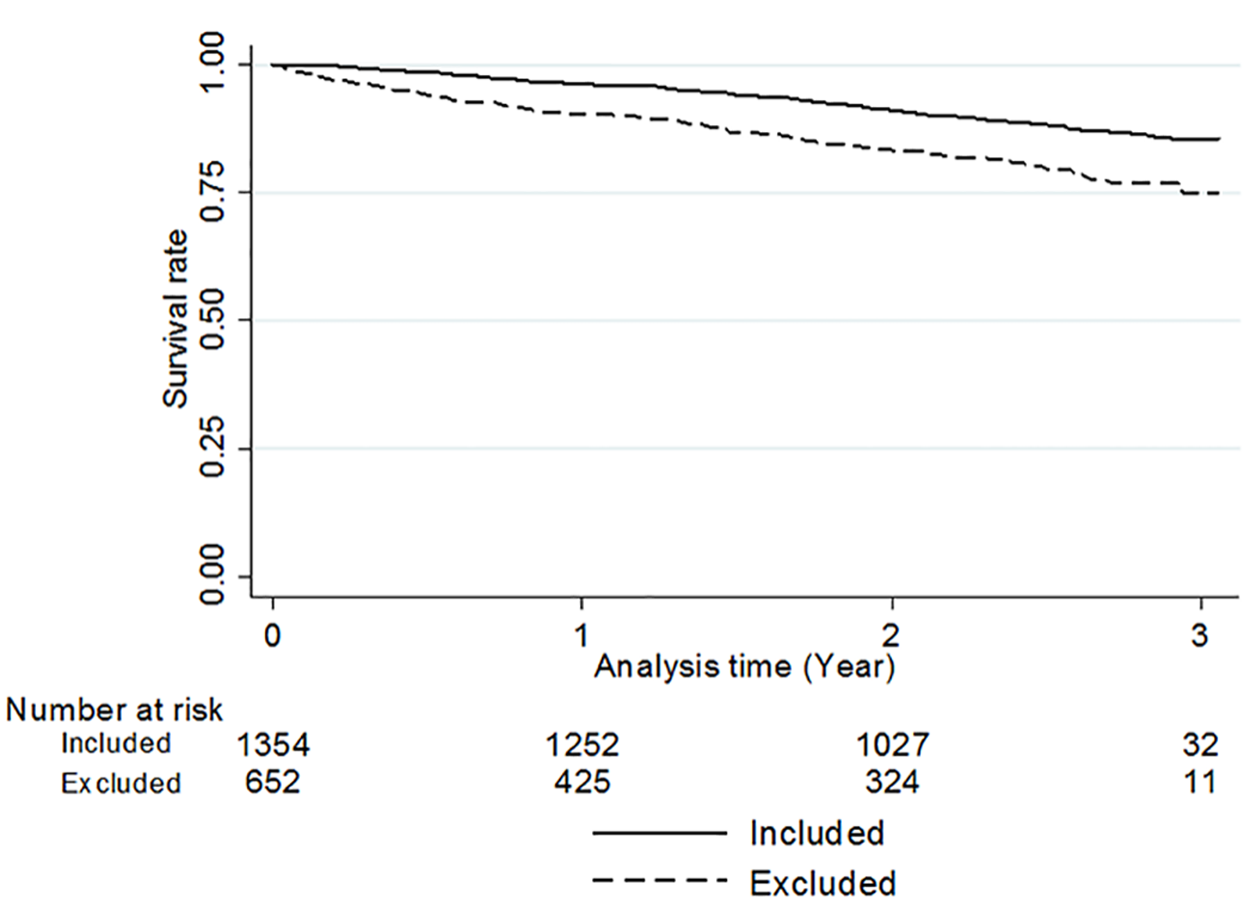
Kaplan-Meier curve for participants included in the final analysis of the primary outcome and excluded participants.

2,742 participants were enrolled in J-DOPPS IV, and 1,354 participants were included in the final analysis for the primary outcome, after excluding 271 participants with time on dialysis less than 3 months and 1,117 participants with missing data in the exposure variable and mortality (Fig 2A). The distribution of each score in CSI-SF is shown in Fig 3. The reliability of this scale was assessed using Cronbach’s alpha, and the values were 0.66 for problem-focused engagement, 0.53 for problem-focused disengagement, 0.64 for emotion-focused engagement, and 0.56 for emotion-focused disengagement. Although the reliabilities of problem-focused disengagement and emotion-focused disengagement were relatively low, those of problem-focused engagement and emotion-focused engagement were moderate. The baseline characteristics of the participants in the final analysis and the excluded participants are summarized in Table 2. Mean (SD) age was 62.9 (11.8) years and median (interquartile range) years of dialysis was 5.4 (1.8–11.5) years in the participants in the final analysis. Several variables, such as age, time on dialysis, diabetes, and depression, tended to be different between the participants in the final analysis and the excluded participants. Baseline characteristics according to group in each subscale of CSI-SF are shown in S1–S4 Tables. The difference in mortality between the participants in the final analysis and the excluded participants was assessed using the Kaplan-Meier curve (Fig 4), which indicates that those in the final analysis showed better survival, though the data on the primary outcome in 465 excluded participants was missing (Log rank test, p-value <0.01).

**Table 2:** Baseline characteristics.

	Participants in the final analysis (n = 1,354)	Participants excluded from the final analysis (n = 1,117)	Number of participants in the final analysis with missing data
Age (years)	62.9 (11.8)	68.1 (12.5)	2
Gender (%; male)	65.4	62.3	0
Years on dialysis	5.4 (1.8–11.5)	4.0 (0.9–9.5)	8
Diabetes (%)	32.2	41.2	15
History of CVD (%)			
CHF	20.3	21.2	9
CAD	30.2	34.4	7
Stroke	13.0	19.9	5
PAD	18.1	22.0	8
Others	29.6	32.1	0
Depression (%)	44.2	47.5	171
Educational status (%; graduated from high school)	91.0	91.4	94
High income (%; ≥5,000,000 yen/year)	38.4	36.3	486
SF-12			
Physical functioning	50 (50–100)	50 (25–75)	
Mental health	62.5 (50–87.5)	62.5 (50–80)	
KDQOL			
Effect of kidney disease	75.0 (59.4–84.4)	70.8 (53.1–83.3)	15
Burden of kidney disease	31.3 (18.8–50.0)	31.3 (12.5–50)	12
Coping strategies			
Engagement			
Problem-focused	12.9 (3.5)		
Emotion-focused	10.3 (3.2)		
Disengagement			
Problem-focused	11.9 (3.3)		
Emotion-focused	10.5 (3.1)		

Note: Values for categorical variables are given as a percentage; values for continuous variables are given as mean (SD) or median (interquartile range)

Abbreviations: CVD, cardiovascular disease; CHF, congestive heart failure; CAD, coronary artery disease; PAD, peripheral artery disease; SD, standard deviation.

### Primary outcome

**Fig 5:**
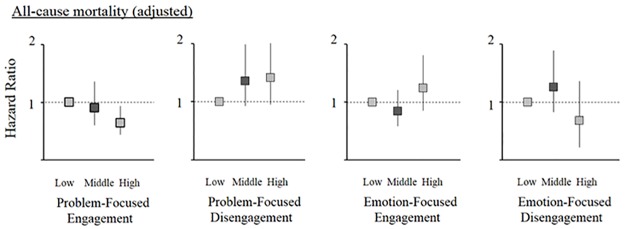
Associations of CSI-SF subscale scores with all-cause mortality. Hazard ratios and 95% confidence intervals in Cox models adjusted for age, gender, years on dialysis, presence of diabetes mellitus, history of cardiovascular disease, depression, effect of kidney disease, burden of kidney disease, educational status and high annual income, are shown with squares and vertical lines, respectively.

During a median (interquartile range) follow-up period of 2.69 (2.07–2.88) years, there were 163 deaths. Censoring occurred for 167 participants: 13 participants for transplantation, 149 participants for transferring to another facility, and 5 participants for other reasons. We estimated HRs for all-cause mortality in comparison with the reference category. HRs in the multivariate Cox models are shown in Fig 5. We found that high problem-focused engagement was significantly associated with better survival (HR 0.64; 95% CI, 0.44 to 0.94). In contrast, none of the other subscales were associated with all-cause mortality.

### Secondary outcome

**Fig 6:**
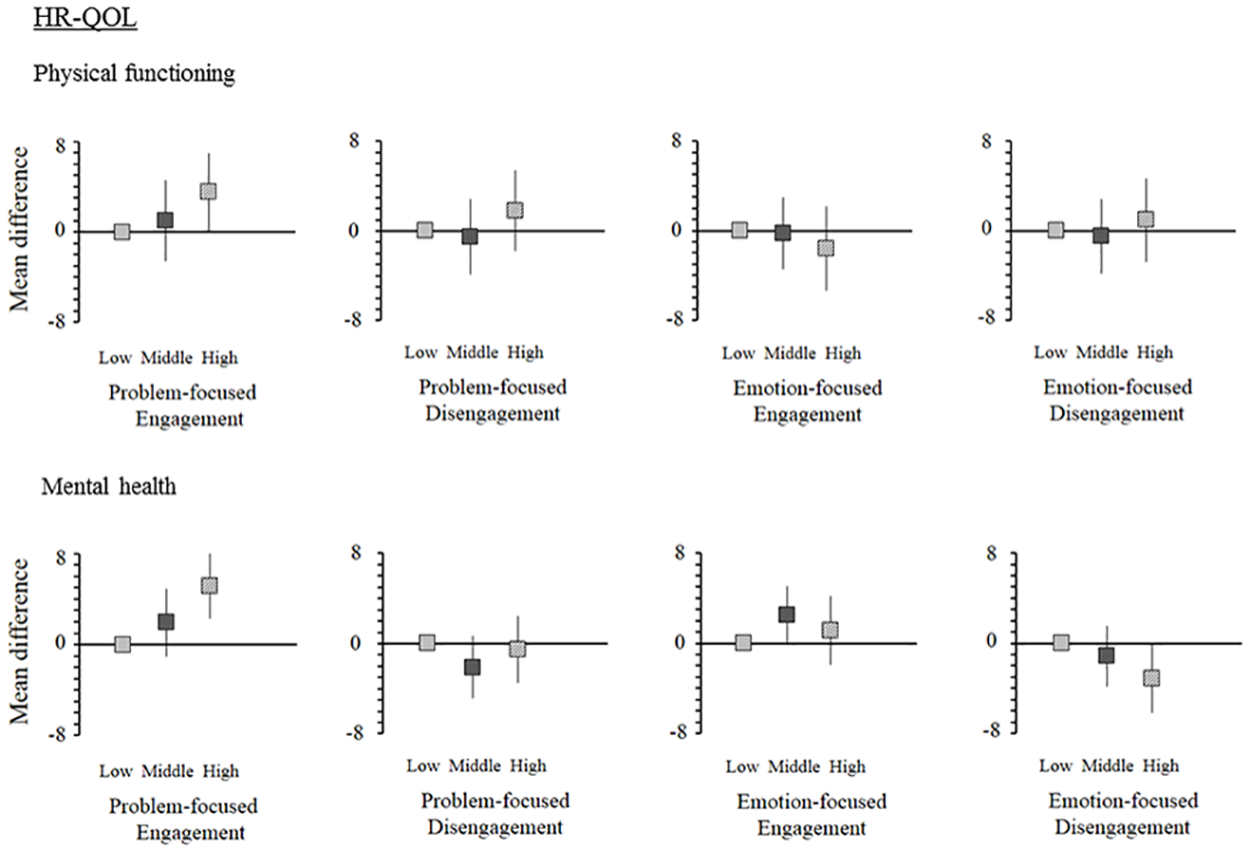
Associations of CSI-SF subscale scores with HR-QOL. Mean differences and 95% confidence intervals in regression models adjusted for age, gender, years on dialysis, presence of diabetes mellitus, history of cardiovascular disease, depression, effect of kidney disease, burden of kidney disease, educational status, high annual income and baseline physical functioning for the change in physical functioning or baseline mental health for the change in mental health are shown with squares and vertical lines, respectively.

After 1,697 participants were excluded because of time on dialysis was less than 3 months or they were missing exposure and outcome data, 1,045 participants were included in the final analysis for the secondary outcome (Fig 2B). Mean differences in the multiple regression model are shown in Fig 6. The multiple regression model showed that high problem-focused engagement was positively associated with change in physical functioning (mean difference, 3.52; 95% CI, 0.07 to 6.96) and mental health (mean difference, 5.17; 95% CI, 2.32 to 8.02). Emotion-focused disengagement was associated only with change in mental health (mean difference, -3.10; 95% CI, -6.19 to -0.01), but not with change in physical functioning (mean difference, 0.95; 95% CI, -2.81 to 4.70). On the other hand, the other subscales were not significantly associated with change in physical functioning and mental health.

### Sensitivity analysis

For the primary outcome, HRs in the multivariate Cox models are shown in Supporting Information. Though not statistically significant, high problem-focused engagement tend to be associated with better survival (HR, 0.75; 95% CI, 0.52 to 1.08) as shown in the primary analysis. For the secondary outcome, mean differences in the multiple regression model are shown in Supporting Information. As shown in the primary analysis, high problem-focused engagement was significantly associated with improvement in physical functioning (mean difference, 3.73; 95% CI, 0.79 to 6.67) and in mental health (mean difference, 5.44; 95% CI, 2.13 to 8.76). In terms of emotion-focused disengagement, it was associated with a non-significant deterioration in mental health (mean difference, -2.23; 95% CI, -5.10 to 0.64), as in the primary analysis.

## Discussion

This is the first longitudinal analysis to investigate the associations of coping strategies with mortality and HR-QOL in hemodialysis patients. First, we found a significant association between problem-focused engagement and longer survival. Second, we found that problem-focused engagement was also associated with improvement in physical functioning and mental health. These results suggest that problem-focused coping may improve survival and HR-QOL in hemodialysis patients.

There are several possible reasons why problem-focused engagement is associated with longer survival. First, problem-focused engagement could be associated with longer survival through improvement in HR-QOL. In the present study, problem-focused engagement was associated with improved HR-QOL in both physical functioning and mental health domains, which were reported to be associated with longer survival[22]. Previous reports about patients with heart failure indicated that problem-focused engagement was associated with better physical and psychological HR-QOL[6]. Problem-focused engagement, including planful problem-solving and cognitive restructuring, can be utilized to alleviate stresses by defining problems and generating alternative solutions. Hemodialysis patients need to deal with stresses related to self-management practices, like fluid or diet restriction. Problem-focused engagement could be useful to alleviate that kind of stress for hemodialysis patients, as well as for patients with heart failure, which could lead to better physical functioning and mental health. Second, a previous study reported that problem-solving coping, which is part of problem-focused engagement, was associated with favorable treatment adherence measured using interdialytic weight gain[2]. Nonadherence in hemodialysis patients, measured using excessive interdialytic gain, skipping treatment, or high serum phosphate levels, was also reported to be associated with mortality in the a previous study[23]. Third, another previous study reported an association between problem-solving coping and reduced recurrence of adverse events following diagnosis of acute coronary syndrome[24]. Cardiovascular disease, including acute coronary syndrome, is the major cause of death in hemodialysis patients, which indicates the possibility that problem-focused engagement is associated with reduced mortality in these patients. In terms of emotion-focused disengagement, which was associated with deterioration in mental health, the result is consistent with previous reports that wishful thinking and self-criticism were associated with increases in anxiety[25]. Another previous study reported that disengagement strategies had a negative effect on psychological HR-QOL in patients with heart failure[6].

Our present results are somewhat inconsistent with the results of a previous study of the association of mortality with coping strategies in patients with ESRD[14]. Although we found no significant associations of mortality with disengagement coping strategies in the present study, a previous study reported that disengagement strategies were associated with mortality in ESRD patients, whereas problem-focused engagement was not significantly associated with mortality[14]. However, this previous study included only 61 veterans with ESRD who were referred to evaluate their indications for renal transplantation, and included ESRD patients receiving peritoneal dialysis and those not receiving dialysis therapy at the baseline evaluation of the study. Additionally, there were only 23 events in this study, which was not enough to adjust for the relevant covariates. Due to the small sample size and differences in the study sample, the result of this previous study may not be comparable with the results of the present study.

The present study has several strengths. First, it is the first report to demonstrate an association between coping strategies and clinical outcomes, including all-cause mortality and HR-QOL, in a longitudinal design with a sample large enough to allow adjustment for relevant confounding factors. Second, external validity was enhanced by the use of random sampling in the recruitment of participants. Third, relevant confounding factors that could affect the association between coping strategies and clinical outcomes were measured in J-DOPPS, and we adjusted for these factors in this analysis.

However, this study has some limitations. First, owing to the observational design, causality could not be assessed. Though baseline characteristics differed among the groups in each subscale of coping strategies (shown in S1–S4 Tables), we adjusted for these relevant confounding factors in the multivariate analysis. The time on dialysis could cause a lead-time bias, though we adjusted for it in the multivariate analysis. However, the results of the present study could be minimally affected by this kind of bias because the coping strategies were not associated with time on dialysis in this sample. This finding seems to be consistent with a previous report saying that coping strategies could be stable through a natural history[7], while also being modifiable using specific interventions[8–10]. Second, the participants were recruited in Japan only and the applicability of the results might accordingly be limited to Japanese. Differences may exist in the use and effect of coping strategies according to cultural and religious background. Although several previous studies have assessed the association of religious coping with other coping strategies, results have been diverse and the association remains unclear[26,27]. Additionally, the associations between religious coping and other coping strategies are reported to differ depending on religiosity[28]. However, Japanese people have less religious belief than people in other countries, such as the United States, Canada, India, Poland, Korea, Slovakia, and Uganda[29], and the associations between coping strategies and clinical outcomes identified in our present study are assumed to be less affected by the participants’ religious backgrounds than in other countries. Third, because of missing data, we excluded the participants without data on exposure and outcome variables in the primary analysis. The baseline characteristics and mortality were different, which could lead to selection bias. To alleviate this bias, we did sensitivity analyses with multiple imputation methods for exposure and outcome variables. Though not statistically significant, problem-focused engagement was associated with longer survival. Furthermore, we found similar associations of problem-focused engagement with improvement of physical functioning and mental health. Fourth, actual social support that could be related to emotion-focused engagement was not measured. However, the association between problem-focused engagement and all-cause mortality or HR-QOL cannot be fully explained by social support because emotion-focused engagement, including social-support coping, was not associated with all-cause mortality or HR-QOL. Finally, the translated version of the CSI-SF we used was not back-translated or validated. However, the translation was approved by the Japanese steering committee of DOPPS, which was blinded to the data analysis. Although the reliabilities of problem-focused disengagement and emotion-focused disengagement were relatively low, the reliability of problem-focused engagement, which was significantly associated with longer survival and QOL improvement, was moderate. We believe that the association was not affected by the translation.

In conclusion, we found that problem-focused engagement is associated with longer survival and improvement in HR-QOL in maintenance hemodialysis patients. These findings have important clinical implications. So far, differences in coping strategies have not been addressed in clinical practice for the maintenance of hemodialysis patients. The finding in the current study suggested that it could be possible to enhance longevity and to improve HR-QOL in hemodialysis patients by modification of problem-focused engagement. Some studies suggested that coping strategies could be modified using cognitive behavioral therapies[8–10]. Interventions, like cognitive behavioral therapies, focusing on problem-focused engagement could be recommended for maintenance hemodialysis patients with impaired problem-focused engagement.

## Supporting information

S1 Fig Associations of CSI-SF subscale scores with all-cause mortality in sensitivity analysis. Hazard ratios and 95% confidence intervals in Cox models adjusted for age, gender, years on dialysis, presence of diabetes mellitus, history of cardiovascular disease, depression, effect of kidney disease, burden of kidney disease, educational status and high annual income are shown with squares and vertical lines, respectively.
(TIF)

S2 Fig Associations of CSI-SF subscale scores with HR-QOL in sensitivity analysis. Mean differences and 95% confidence intervals in regression models adjusted for age, gender, years on dialysis, presence of diabetes mellitus, history of cardiovascular disease, depression, effect of kidney disease, burden of kidney disease, educational status, high annual income and baseline physical functioning for the change in physical functioning or baseline mental health for the change in mental health, are shown with squares and vertical lines, respectively.
(TIF)

S1 Table Baseline characteristics according to groups in problem-focused engagement. (DOCX)

S2 Table Baseline characteristics according to groups in emotion-focused engagement. (DOCX)

S3 Table Baseline characteristics according to groups in problem-focused disengagement. (DOCX)

S4 Table Baseline characteristics according to groups in emotion-focused disengagement. (DOCX)
