# Supplementary figures and images for: Association of coping strategies with mortality and health-related quality of life in hemodialysis patients: The Japan Dialysis Outcomes and Practice Patterns Study
Source: PLoS One. 2017 Jul 25;12(7):e0180498. doi: 10.1371/journal.pone.0180498 (PMC5526523; doi:10.1371/journal.pone.0180498)

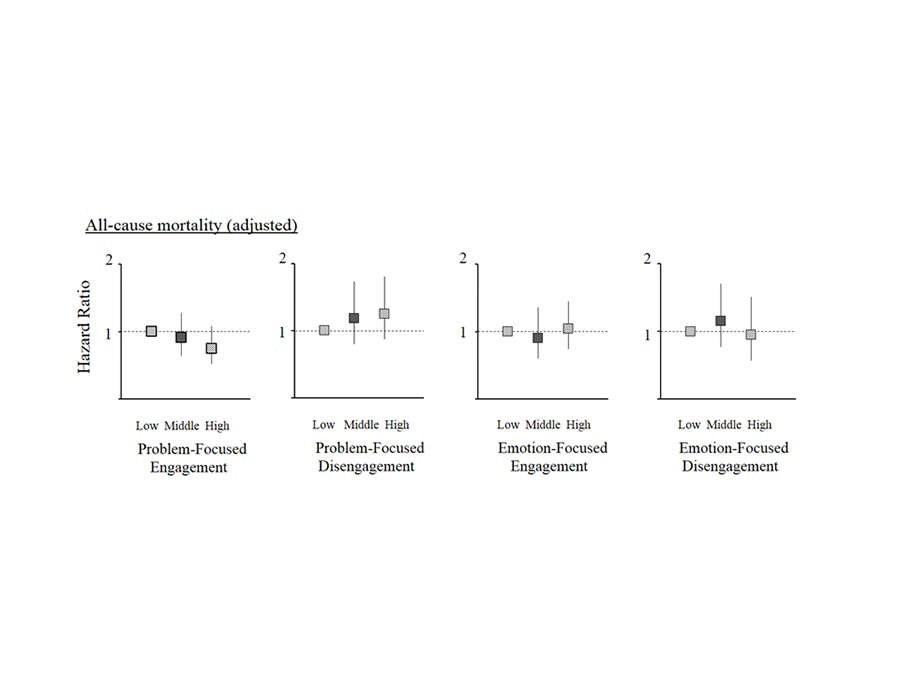

Supplement: S1 Fig — Hazard ratios and 95% confidence intervals in Cox models adjusted for age, gender, years on dialysis, presence of diabetes mellitus, history of cardiovascular disease, depression, effect of kidney disease, burden of kidney disease, educational status and high annual income are shown with squares and vertical lines, respectively. (TIF) [file pone.0180498.s001.tif]

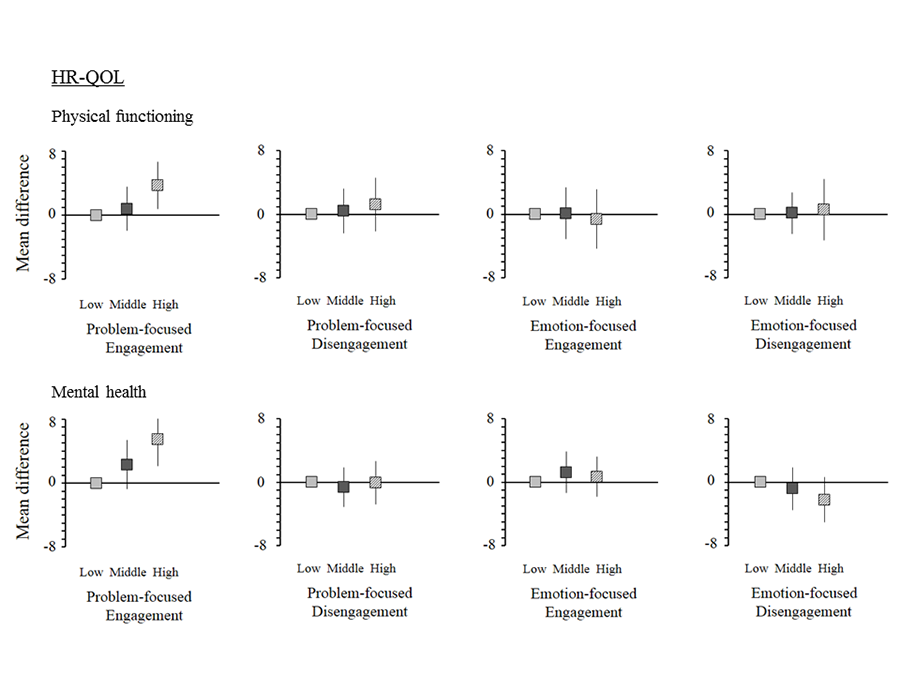

Supplement: S2 Fig — Mean differences and 95% confidence intervals in regression models adjusted for age, gender, years on dialysis, presence of diabetes mellitus, history of cardiovascular disease, depression, effect of kidney disease, burden of kidney disease, educational status, high annual income and baseline physical functioning for the change in physical functioning or baseline mental health for the change in mental health, are shown with squares and vertical lines, respectively. (TIF) [file pone.0180498.s002.tif]
