# Supplementary material for: Association of coping strategies with mortality and health-related quality of life in hemodialysis patients: The Japan Dialysis Outcomes and Practice Patterns Study
Source: PLoS One. 2017 Jul 25;12(7):e0180498. doi: 10.1371/journal.pone.0180498 (PMC5526523; doi:10.1371/journal.pone.0180498)
Supplement: S1 Table — (DOCX) [file pone.0180498.s003.docx]

**S1 Table. Baseline characteristics according to groups in problem-focused engagement**

|  | Low group  (n=615) | Middle group  (n=300) | High group  (n=439) |
| --- | --- | --- | --- |
| Score of PFE | 4-12 | 13-14 | 15-20 |
| Age (years) | 63.4 (11.8) | 61.8 (11.4) | 62.9 (12.0) |
| Gender (%; male) | 65.4 | 62.7 | 67.4 |
| Years on dialysis | 5.9 (1.9-11.8) | 5.2 (1.7-11.5) | 4.8 (1.9-10.8) |
| Diabetes (%) | 36.4 | 29.0 | 28.5 |
| History of CVD (%) |  |  |  |
| CHF | 21.2 | 17.2 | 21.2 |
| CAD | 32.7 | 27.1 | 28.8 |
| Stroke | 14.0 | 10.3 | 13.3 |
| PAD | 18.3 | 16.4 | 18.9 |
| Others | 30.3 | 26.3 | 30.7 |
| Depression (%) | 59.4 | 40.6 | 26.8 |
| Educational status  (%; graduated from  high school) | 89.6 | 91.3 | 92.7 |
| High income  (%; ≥5,000,000 yen/year) | 38.8 | 39.0 | 37.4 |
| KDQOL |  |  |  |
| Effect of kidney disease | 71.4 (53.1- 81.3) | 75.0 (59.4- 84.4) | 78.1 (65.6- 87.5) |
| Burden of kidney disease | 25.0 (12.5- 43.8) | 31.3 (18.8- 50) | 37.5 (25.0- 56.3) |

Note: Values for categorical variables are given as a percentage; values for continuous variables are given as mean (SD) or median (interquartile range) except for score of PFE. Values for PFE are given as a range.

Abbreviations: PFE, problem-focused engagement; CVD, cardiovascular disease; CHF, congestive heart failure; CAD, coronary artery disease; PAD, peripheral artery disease; SD, standard deviation.
