# Supplementary material for: Association of coping strategies with mortality and health-related quality of life in hemodialysis patients: The Japan Dialysis Outcomes and Practice Patterns Study
Source: PLoS One. 2017 Jul 25;12(7):e0180498. doi: 10.1371/journal.pone.0180498 (PMC5526523; doi:10.1371/journal.pone.0180498)
Supplement: S2 Table — (DOCX) [file pone.0180498.s004.docx]

**S2 Table. Baseline characteristics according to groups in emotion-focused engagement**

|  | Low group  (n=558) | Middle group  (n=477) | High group  (n=319) |
| --- | --- | --- | --- |
| Score of EFE | 4-9 | 10-12 | 13-20 |
| Age (years) | 63.1 (11.2) | 62.9 (11.4) | 62.5 (12.6) |
| Gender (%; male) | 71.7 | 65.8 | 53.9 |
| Years on dialysis | 4.9 (2.0-11.2) | 6.1 (1.8-11.8) | 5.1 (1.7-11.4) |
| Diabetes (%) | 31.7 | 28.8 | 38.1 |
| History of CVD (%) |  |  |  |
| CHF | 18.4 | 19.8 | 24.4 |
| CAD | 30.8 | 31.3 | 27.5 |
| Stroke | 12.2 | 12.6 | 14.8 |
| PAD | 17.5 | 18.1 | 19.0 |
| Others | 31.5 | 28.3 | 28.2 |
| Depression (%) | 43.0 | 44.3 | 46.1 |
| Educational status  (%; graduated from  high school) | 90.9 | 92.1 | 89.2 |
| High income  (%; ≥5,000,000 yen/year) | 39.8 | 38.9 | 35.2 |
| KDQOL |  |  |  |
| Effect of kidney disease | 75.0 (62.5- 85.7) | 71.9 (56.3- 84.4) | 75.0 (57.7- 84.4) |
| Burden of kidney disease | 31.3 (18.8- 50.0) | 31.3 (18.8- 50.0) | 31.3 (12.5- 43.8) |

Note: Values for categorical variables are given as a percentage; values for continuous variables are given as mean (SD) or median (interquartile range) except for score of EFE. Values for EFE are given as a range.

Abbreviations: EFE, emotion-focused engagement; CVD, cardiovascular disease; CHF, congestive heart failure; CAD, coronary artery disease; PAD, peripheral artery disease; SD, standard deviation.
