# Supplementary material for: Association of coping strategies with mortality and health-related quality of life in hemodialysis patients: The Japan Dialysis Outcomes and Practice Patterns Study
Source: PLoS One. 2017 Jul 25;12(7):e0180498. doi: 10.1371/journal.pone.0180498 (PMC5526523; doi:10.1371/journal.pone.0180498)
Supplement: S3 Table — (DOCX) [file pone.0180498.s005.docx]

**S3 Table. Baseline characteristics according to groups in problem-focused disengagement**

|  | Low group  (n=469) | Middle group  (n=476) | High group  (n=409) |
| --- | --- | --- | --- |
| Score of PFD | 4-10 | 11-13 | 14-20 |
| Age (years) | 63.3 (10.9) | 62.0 (12.1) | 63.5 (12.3) |
| Gender (%; male) | 74.2 | 65.3 | 55.5 |
| Years on dialysis | 5.6 (1.8-11.3) | 5.4 (1.6-12.1) | 5.2 (2.1-11.4) |
| Diabetes (%) | 35.3 | 30.0 | 31.2 |
| History of CVD (%) |  |  |  |
| CHF | 19.4 | 19.3 | 22.6 |
| CAD | 32.9 | 30.7 | 26.5 |
| Stroke | 12.0 | 12.6 | 14.5 |
| PAD | 17.4 | 18.8 | 17.9 |
| Others | 29.2 | 30.0 | 29.6 |
| Depression (%) | 44.2 | 45.7 | 42.5 |
| Educational status  (%; graduated from  high school) | 91.6 | 90.5 | 90.8 |
| High income  (%; ≥5,000,000 yen/year) | 40.8 | 37.5 | 36.8 |
| KDQOL |  |  |  |
| Effect of kidney disease | 75.0 (62.5- 84.4) | 75.0 (58.3- 84.4) | 71.9 (56.3- 84.4) |
| Burden of kidney disease | 37.5 (18.8- 50.0) | 31.3 (18.8- 50.0) | 31.3 (12.5- 43.8) |

Note: Values for categorical variables are given as a percentage; values for continuous variables are given as mean (SD) or median (interquartile range) except for score of PFD. Values for PFD are given as a range.

Abbreviations: PFD, problem-focused disengagement; CVD, cardiovascular disease; CHF, congestive heart failure; CAD, coronary artery disease; PAD, peripheral artery disease; SD, standard deviation.
