# Supplementary material for: Association of coping strategies with mortality and health-related quality of life in hemodialysis patients: The Japan Dialysis Outcomes and Practice Patterns Study
Source: PLoS One. 2017 Jul 25;12(7):e0180498. doi: 10.1371/journal.pone.0180498 (PMC5526523; doi:10.1371/journal.pone.0180498)
Supplement: S4 Table — (DOCX) [file pone.0180498.s006.docx]

**S4 Table. Baseline characteristics according to groups in emotion-focused disengagement**

|  | Low group  (n=515) | Middle group  (n=508) | High group  (n=331) |
| --- | --- | --- | --- |
| Score of EFD | 4-9 | 10-12 | 13-20 |
| Age (years) | 64.6 (11.6) | 62.3 (11.1) | 61.2 (12.9) |
| Gender (%; male) | 67.8 | 66.7 | 59.8 |
| Years on dialysis | 5.4 (1.8-11.3) | 5.3 (1.7-11.7) | 5.5 (2.1-11.7) |
| Diabetes (%) | 34.1 | 31.9 | 29.8 |
| History of CVD (%) |  |  |  |
| CHF | 22.6 | 18.9 | 18.9 |
| CAD | 34.8 | 27.6 | 27.1 |
| Stroke | 13.7 | 12.0 | 13.4 |
| PAD | 18.2 | 19.5 | 15.5 |
| Others | 31.7 | 28.4 | 28.4 |
| Depression (%) | 34.0 | 46.1 | 57.2 |
| Educational status  (%; graduated from  high school) | 90.2 | 91.5 | 91.2 |
| High income  (%; ≥5,000,000 yen/year) | 42.6 | 41.0 | 27.4 |
| KDQOL |  |  |  |
| Effect of kidney disease | 75 (62.5- 85.7) | 75 (59.4- 84.4) | 68.8 (53.6- 81.3) |
| Burden of kidney disease | 37.5 (18.8- 50) | 31.3 (18.8- 50) | 25 (12.5- 43.8) |

Note: Values for categorical variables are given as a percentage; values for continuous variables are given as mean (SD) except for score of EFD. Values for EFD are given as a range.

Abbreviations: EFD, emotion-focused disengagement; CVD, cardiovascular disease; CHF, congestive heart failure; CAD, coronary artery disease; PAD, peripheral artery disease; SD, standard deviation.
